# Residential Relocation by Older Adults in Response to Incident Cardiovascular Health Events: A Case-Crossover Analysis

**DOI:** 10.1155/2014/951971

**Published:** 2014-03-23

**Authors:** Gina S. Lovasi, John M. Richardson, Carlos J. Rodriguez, Willem J. Kop, Ali Ahmed, Arleen F. Brown, Heather Greenlee, David S. Siscovick

**Affiliations:** ^1^Department of Epidemiology, Columbia University, 722 West 168th Street, New York, NY 10032, USA; ^2^Department of Medicine, Medical Center Boulevard, Winston Salem, NC, USA; ^3^Department of Medical Psychology and Neuropsychology, Tilburg University, Warandelaan 2, Tilburg, NL, USA; ^4^Department of Medicine, University of Alabama, 1720 2nd Avenue South, Birmingham, AL, USA; ^5^Section of Geriatrics, Birmingham VA Medical Center, 700 19th Street South, Birmingham, AL 35233, USA; ^6^Department of Medicine, University of California, 911 Broxton Avenue, Los Angeles, CA, USA; ^7^Departments of Medicine and Epidemiology, University of Washington, 325 Ninth Avenue, Seattle, WA, USA

## Abstract

*Objective*. We use a case-crossover analysis to explore the association between incident cardiovascular events and residential relocation to a new home address. *Methods*. We conducted an ambidirectional case-crossover analysis to explore the association between incident cardiovascular events and residential relocation to a new address using data from the Cardiovascular Health Study (CHS), a community-based prospective cohort study of 5,888 older adults from four U.S. sites beginning in 1989. Relocation was assessed twice a year during follow-up. Event occurrences were classified as present or absent for the period preceding the first reported move, as compared with an equal length of time immediately prior to and following this period. *Results*. Older adults (65+) that experience incident cardiovascular disease had an increased probability of reporting a change of residence during the following year (OR 1.6, 95% confidence interval (CI) = 1.2–2.1). Clinical conditions associated with relocation included stroke (OR: 2.0, 95% CI: 1.2–3.3), angina (OR: 1.6, 95% CI: 1.0–2.6), and congestive heart failure (OR: 1.5, 95% CI: 1.0–2.1). *Conclusions*. Major incident cardiovascular disease may increase the probability of residential relocation in older adults. Case-crossover analyses represent an opportunity to investigate triggering events, but finer temporal resolution would be crucial for future research on residential relocations.

## 1. Introduction

Case-crossover designs [1] offer the possibility of investigating time-varying exposures as triggering subsequent events, while allowing each person to serve as his or her own control. A case-crossover design may be useful for studying a transient increase in the risk of experiencing an acute-onset event. We illustrate the potential relevance of this design to research on neighborhoods and health by considering the temporal association between incident cardiovascular disease events and residential relocation to a new home address.

Residential relocation by older adults may be considered a consequence of disease or disability [2] that could be addressed as part of a public health strategy for tertiary prevention because it is likely to be followed by further declines in physical health and quality of life. Among older adults, changes in residence including moves to downsize, to be closer to family, or into specialized settings such as nursing homes, may adversely affect quality of life [3, 4]. The anticipation of a move has also been associated with depression [5], suggesting that the move itself could have negative consequences for health and wellbeing. Yet the possibility of confounding (e.g., insufficient economic resources contributing to disease risk and to a less stable residential environment) or reverse causation (e.g., severity of health problem underlies both the necessity for relocation and the subsequent declines) makes interpretation of cross-sectional associations difficult. Further, even for older adults who prefer to age in place, the relocation to a new residential address may represent an improvement in access to health care resources or instrumental social support relevant to managing chronic conditions.

We used the Cardiovascular Health Study (CHS) to explore the triggering effect of negative health events on residential relocations. Longitudinal data were analyzed in a case-crossover framework by comparing each of the individuals to themselves across different time periods. We hypothesized that older adults who have recently experienced the onset of clinical cardiovascular disease, defined as myocardial infarction (MI), angina, stroke, congestive heart failure (CHF), claudication, or transient ischemic attack (TIA), will have an increased probability of moving to a new residential address during the following months. While we would expect major clinical or functional events to predict residential relocations, we also hypothesized that those with limited resources (socioeconomically disadvantaged or isolated older adults) will be less able to adapt to new health limitations in their current environment, and thus more likely to respond to poor health by relocating. This analysis represents the first attempt to use the case-crossover methodology to assess the impact of disease onset on residential relocation.

## 2. Materials and Methods

### 2.1. Study Population

The Cardiovascular Health Study (CHS) is a prospective, population-based cohort study of risk factors for cardiovascular disease and stroke in 5,888 adults 65 years or older at baseline [6]. The original cohort of 5,201 participants was recruited in 1989-1990, and additional 687 African Americans were enrolled in 1992-1993. At study entry, all CHS participants were noninstitutionalized older adults who expected to remain in the area for at least three years. Health data, including hospitalizations, functional status, and adjudicated cardiovascular disease events, were assessed throughout follow-up as were the changes of residence.

### 2.2. Incident Cardiovascular Health Events

The potential triggers of interest were definite or probable incident cardiovascular disease events which included myocardial infarction (MI), angina, stroke, transient ischemic attack (TIA), congestive heart failure (CHF), or claudication. All outcome events were adjudicated by the CHS Events Subcommittee as described previously [7]. Cardiovascular events were ascertained via biannual phone surveillance calls with participants (or proxies) to identify self-reported events and all hospitalizations and by surveying discharge lists from local hospitals and death certificates from state vital statistics offices for potential cardiovascular events. All reports were investigated. Hospital records were obtained and abstracted. All cardiovascular events were validated by a committee of physicians using standardized criteria. Cases were classified as formal events after the committee performed a review of medical records and an adjudication process.

### 2.3. Residential Relocation Assessment

Participant relocation to a new home address was the main outcome of interest and was assessed at baseline and then twice a year during most of the follow-up period through 2006. There was, however, a four-year period between years five and nine when participant moves were only assessed once each year. Participants were asked whether they moved in the last six months, and if they had moved in the last six months, they were asked whether they had moved within the last month. Data for the six-month move is complete for 97% of available respondents across all move assessments. Data for the one-month move is complete for 87% of those reporting any move in the past 6 months. For the case-crossover analysis, only the first reported move of those participants who had moved during the study time was included. This restriction was intended to simplify the analyses, and to focus attention on moving away from the home where the participants lived at baseline and where they intended to stay for at least 3 years; information was not available on whether subsequent residential locations were intended to be long-term or temporary.

### 2.4. Potential Effect Modifiers

Participant demographic characteristics and self-reported health status were collected at baseline. Neighborhood poverty was assessed at the census block group level, based on the proportion of residents living below the federal poverty line. Social support was assessed using a reduced, six-question version of the International Support Evaluation List [8].

### 2.5. Statistical Analysis

Because of our focus on the relative timing of health changes and residential moves and an interest in controlling for potential individual-level confounders including unmeasured aspects of residential history, we employed a case-crossover design [1]. The case-crossover design uses time-varying data on outcome occurrences and their potential triggers, so that each person serves as his or her own control. A “trigger” in this case can be thought of as a final component cause leading a susceptible person to experience an event. The case-crossover design was used to assess whether older adults who had recently experienced an incident cardiovascular disease event were more likely to report a move than those same individuals at another time. The case-crossover approach has the advantage of eliminating confounding by stable personal characteristics, and allowing for explicit variations in the window of exposure. The case-crossover analysis was restricted to CHS participants who reported at least one change in residence during follow-up.

**Figure 1:**
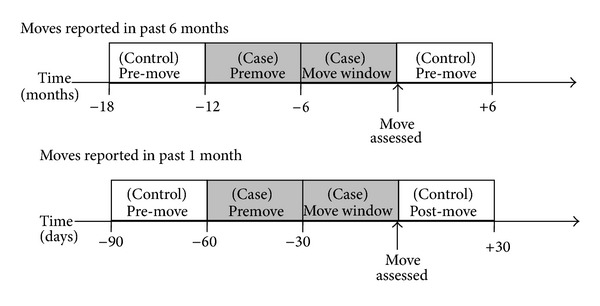


Precision on the timing of moves was limited. Participants reported whether a move occurred within a 6 month window, and whether they had moved in the most recent 1 month window for the subset of participants who reported moving in the past six months. We reasoned that an event occurring during or shortly prior to that move window could have triggered the move, and thus identified a 12 month “case” window for the 6 month analysis (Figure 1). We used control periods both before and after the residential relocation was reported because the biases for these two periods are expected to be different [9, 10]. Ideally, the control windows should reflect the typical level of exposure experienced while at risk for the outcome; in our analysis, we wanted to select control windows that would represent the probability of experiencing incident cardiovascular disease events around the time when each participant was at risk for reporting their first move to a new residential address during follow-up. Since cardiovascular risk increases with age, selecting a control window that was always before the case window would tend to bias results towards supporting the hypothesis that more CVD events occurred in the year before a first move was reported. The pre- and postmove “control” time periods were from 12 to 18 months before the move was reported and from 0 to 6 months after a move was reported (Figure 1). To ensure that all the case and control windows occurred within the CHS follow-up period, any move occurring in the first eighteen months after enrollment was excluded from the analysis (*N* = 353). For analyses of moves occurring in the past 1 month, the case period consisted of the 60 days before a move was reported, and control periods were the month immediately before that (61–90 days before the move assessment) plus the month following the move assessment (0 to 30 days after the move was reported) (Figure 1).

Conditional logistic regression was used to compare the incidence of CVD events during the case and control time periods, relative to the timing of the first move. Odds ratios greater than 1.00 for case windows were considered to support the hypothesis that cardiovascular events may have triggered moving to a new residence. Multinomial logistic regression was also used to explore the pattern across multiple case and control time periods.

By design, case-crossover analysis controls for stable individual covariates and age-adjustment did not change the observed patterns, so unadjusted odds ratios are presented. Stratified analyses were conducted to evaluate effect modification by age, sex, race, marital status, income, neighborhood poverty rate, social support, and self-reported health status at baseline, with interaction *P* values based on a Wald test of interaction terms added to the main analysis.

## 3. Results

Over fifteen hundred (1,564) participants reported moving to a new home address at least once during follow-up. Overall, residential relocation during the course of follow-up was more likely to be reported by participants who were female, non-Black, not married/separated, lower income, in better health at baseline, and with more years of CHS follow-up (Table 1). The first reported move occurred on average 8.1 years (median: 8.4) after baseline.

Comparing the case and control time periods, the odds of any event were higher in the case time periods (Table 2), suggesting that cardiovascular events were more likely to occur around the time of the first reported residential relocation. The odds ratio for an incident CVD event was highest for the time window when the move occurred (odds ratio: 2.76; 95% confidence interval: 1.98 to 3.84). For a subset of 460 participants, the first residential relocation was identified as occurring within the past month, allowing the timing of the move to be defined more precisely. The odds of a cardiovascular event were higher immediately before or during the one month period when a move reportedly occurred (Table 3).

Among the specific adjudicated outcomes considered, stroke had the largest odds ratio but all confidence intervals overlapped (Table 4). Stratified analyses revealed no statistically significant interactions with age, sex, race, marital status, income, neighborhood poverty, or baseline health status (Table 5). However, there were trends for the odds ratios to be larger for participants who were female or black. The only statistically significant effect modification observed was for social support, and this was in the opposite of the hypothesized direction such that the odds ratio was larger for those reporting more social support. Because of small sample size, the results for specific outcomes and stratified analyses are not presented for the one-month move window analyses.

## 4. Discussion

The findings potentially support our hypothesis that major cardiovascular health events play a role in triggering residential relocation moves, but they do not allow us to rule out the possibility of a bidirectional relationship in which residential relocation moves also trigger cardiovascular disease. CVD events were more likely to occur both during the 6 or 1 month time window when the move occurred and the window immediately preceding the move, as compared to the same individuals' CVD event risk during previous or subsequent control windows.

The power to examine effect modification was limited, but trends suggested that the temporal association of incident cardiovascular events with subsequent relocation was stronger among participants with more baseline social support. This was in the opposite direction of our hypothesized effect modification. Perhaps social support may help to make relocation possible for some older adults. Qualitative investigation into the process and experience of relocation could help to elucidate these pathways.

This study illustrates a case-crossover approach and contributes to a previous literature describing health challenges as among the reasons for older adults to move to a new home address [5, 11, 12]. Other key considerations for relocation include regional climate [13] and social identity [4, 5]. Although previous work has described the pattern of increasing probability of residential relocation at later ages [3] or with increasing health impairment generally [5], we are not aware of other studies quantifying the role of incident cardiovascular disease as a trigger for relocation in a population-based sample.

The potential for older adults to move to a new home in response to health problems also represents a source of bias in cross-sectional or short-term longitudinal studies of neighborhood effects on disease or disability later in life. Studies of older adults have investigated the health effects of neighborhood socioeconomic status [14–16], neighborhood problems [17], cohesive or supportive social environments [18, 19], physical activity promoting features [18, 20–26], and food stores [22, 27]. These associations may be biased if previous health problems played a role in determining both residential location and health at the time of study participation. Indeed, if supportive neighborhood features allow individuals to remain in their homes as their physical functioning declines, these same features might be associated with* more* disability in cross-sectional studies of noninstitutionalized populations. This is likely to result in bias towards the null or in the direction opposite to the hypothesized association.

### 4.1. Strengths and Limitations

A key strength of this analysis was the use of a population-based cohort with well-characterized CVD events over an extended follow-up period. Also, the case-crossover methodology addressed confounding by stable characteristics at the individual level, including aspects of prebaseline residential and health history that were not measured.

There were also several important limitations to this analysis. Data on residential relocations was self-reported, without details on timing or reason for moving. Statistical power to examine the moves with more specific timing (within the past month) or the hypotheses regarding effect modification were limited. Some bias towards the null may have been caused by relocations due to worsening risk factors or subclinical disease prior to the recorded health event or delayed effects of the health decline. Bias away from the null may have been caused by time-varying confounders such as bereavement, which could contribute to both CVD and residential relocations. Finally, we have considered only a subset of the health conditions that could potentially contribute to residential relocation, and future work should consider non-CVD health problems, such as fractures, that may lead to functional impairment and residential relocation.

### 4.2. Conclusions

In this population-based study with more than a decade of follow-up, we observed that cardiovascular events tend to occur before or close to the time of moving to a new home. Future work exploring the reasons for and timing of moves in response to poor health among older adults should integrate more detailed information on the timing and nature of each move. Further research on how residential relocations, living arrangements, and broader neighborhood environments affect health may help to identify opportunities to support management of chronic health conditions and preserve quality of life, but the threat of reverse causation bias should also be considered. Future work should examine the postevent living arrangements of individuals with CVD and consider how multiple longitudinal designs including case-crossover analysis can inform our understanding of both susceptibility and triggers.

## Figures and Tables

**Table 1:** Baseline sample characteristics.

	Moved	Did not move	*P* value
	*N* = 1,564	*N* = 3,971	
	% or mean (SD)	% or mean (SD)	
Age			
Years	72.9 (5.5)	72.7 (5.6)	0.160
Sex			
Male	33%	46%	0.000
Female	67%	54%	
Race			
Non-Black	87%	84%	0.004
Black	13%	16%	
Marital status			
Married	62%	69%	0.000
Not-married/separated	38%	31%	
Income group			
Under $5,000	5%	5%	0.003
$5,000 to $7,999	12%	9%	
$8,000 to $11,999	13%	12%	
$12,000 to $15,999	14%	16%	
$16,000 to $24,999	20%	20%	
$25,000 to $34,999	14%	16%	
$35,000 to $49,999	11%	10%	
Over $50,000	11%	13%	
Neighborhood poverty rate			
Poverty rate at or above median	51%	50%	0.367
Poverty rate below median	49%	50%	
Social support score			
At or higher than median	51%	50%	0.248
Lower than median	49%	50%	
General health			
Excellent/very good	43%	38%	0.000
Good/fair/poor	57%	62%	
Length of CHS follow-up			
Years			

**Table 2:** Case-crossover analysis of cardiovascular event timing relative to a six-month period during which participants reported their first residential relocation (*n* = 1,564).

Reference group: control	Event count	OR	*P* value	(95% CI)	
Control	159	1 (Ref.)			
Case	213	**1.63**	**0.000**	**1.24**	**2.13**
Premove control	65	1 (Ref.)			
Premove case	92	**1.53**	**0.018**	**1.08**	**2.17**
Move-window case	144	**2.76**	**0.000**	**1.98**	**3.84**
Postmove control	103	**1.76**	**0.001**	**1.25**	**2.49**

Notes: OR: indicates odds ratio from a conditional logistic or multinomial regression, and OR values greater than 1.00 indicate that cardiovascular events were disproportionately occurring before or during the 6-month time window when a residential relocation was reported to have occurred; CI: indicates confidence interval; bold face: is used to indicate statistical significance (*P* < 0.05).

**Table 3:** Case-crossover analysis of any cardiovascular event relative to timing of a one-month move window (*n* = 460).

Reference group: control	Event count	OR	*P* value	(95% CI)	
Control	16	1 (Ref.)			
Case	28	**2.00**	**0.050**	**1.00**	**4.00**
Premove control	12	1 (Ref.)			
Premove case	16	1.35	0.444	0.63	2.88
Move-window case	15	1.26	0.559	0.58	2.72
Postmove control	4	0.33	0.055	0.10	1.02

Notes: OR: indicates odds ratio from a conditional logistic or multinomial regression, and OR values greater than 1.00 indicate that cardiovascular events were disproportionately occurring before or during the 1-month time window when a residential relocation was reported to have occurred; CI: indicates confidence interval; bold face: is used to indicate statistical significance (*P* < 0.05).

**Table 4:** Case-crossover analysis of cardiovascular event timing relative to a six-month period during which participants reported their first residential relocation (*n* = 1,564).

Reference group: control		Event count	OR	*P* value	(95% CI)	
Any event	Control	159	1 (Ref.)			
	Case	213	**1.63**	**0.000**	**1.24**	**2.13**
MI	Control	22	1 (Ref.)			
	Case	32	1.53	0.152	0.86	2.72
Angina	Control	43	1 (Ref.)			
	Case	61	**1.62**	**0.041**	**1.02**	**2.57**
Stroke	Control	32	1 (Ref.)			
	Case	55	**2.00**	**0.007**	**1.21**	**3.30**
TIA	Control	10	1 (Ref.)			
	Case	11	1.1	0.827	0.47	2.59
CHF	Control	90	1 (Ref.)			
	Case	113	**1.46**	**0.039**	**1.02**	**2.09**
Claudication	Control	16	1 (Ref.)			
	Case	14	0.82	0.655	0.34	1.97

Notes: OR: indicates odds ratio from a conditional logistic regression and OR values greater than 1.00 indicate that cardiovascular events were disproportionately occurring before or during the 6-month time window when a residential relocation was reported to have occurred; CI: indicates confidence interval; MI: indicates myocardial infarction; CHF: indicates congestive heart failure; TIA: indicates transient ischemic attack; procedures included coronary artery bypass or angioplasty; bold face: is used to indicate statistical significance (*P* < 0.05).

**Table 5:** Stratified case-crossover analysis of cardiovascular event timing relative to a six-month period during which participants reported their first residential relocation.

	OR	95% CI		Interaction *P* value
Age at baseline				
Mean age (73) and older	1.54	1.07	2.24	0.895
72 and younger	1.49	0.97	2.28	
Sex				
Male	1.23	0.81	1.90	0.111
Female	1.93	1.37	2.74	
Race				
Non-black	1.51	1.13	2.00	0.134
Black	3.00	1.28	7.06	
Marital status at baseline				
Married	1.65	1.15	2.36	0.928
Not married/separated	1.61	1.07	2.40	
Income at baseline				
At or above $16,000	1.65	1.10	2.48	0.853
Below $16,000	1.74	1.19	2.54	
Neighborhood poverty at baseline				
Poverty rate at or above median	1.68	1.13	2.52	0.910
Poverty rate below median	1.63	1.07	2.48	
Social support score at baseline				
At or stronger than median	2.09	1.38	3.17	0.041
Weaker than median	1.15	0.78	1.71	
General health at baseline				
Excellent/very good	1.26	0.79	2.02	0.334
Good/fair/poor	1.68	1.18	2.38	

Notes: OR: indicates odds ratio from a conditional logistic regression and OR values greater than 1.00 indicate that cardiovascular events were disproportionately occurring before or during the 6-month time window when a residential relocation was reported to have occurred; CI: indicates confidence interval.
